# Primary non-Hodgkin lymphoma of the prostate: a case report

**DOI:** 10.3332/ecancer.2017.789

**Published:** 2017-12-12

**Authors:** Oscar D Martín, Luis Alfredo Wadskier, Yesica Quiroz, Heilen P Bravo, Giovanni Cacciamani, Paola Umaña, Luis Medina

**Affiliations:** 1 Universidad Cooperativa de Colombia, Facultad de Medicina, Departamento de Investigación -GRIVI, Villavicencio, Colombia; 2 Fundación Universitaria de Ciencias de la Salud, Hospital de San José, Servicio de Urología Bogotá, Colombia; 3 Department of Urology, University of Verona, Italy; 4 Fundación Universitaria de Ciencias de La Salud, Hospital de San José, Servicio de Hemato-Oncología Bogotá, Colombia

**Keywords:** prostate, non-Hodgkin lymphoma, extra-nodal marginal zone-B lymphoma

## Abstract

This report is of a 68-year-old male patient with a three-year history of severe, progressive, low urinary tract symptoms (LUTS) with a score of 20 points on the International Symptom Scale. The patient received alpha-1-blocker therapy without adequate response. Transurethral resection of the prostate was performed, and the anatomopathological report indicated the presence of a haematolymphoid small-cell neoplasia and glandulostromal prostatic hyperplasia. Posterior immunohistochemistry evaluation reported an extra-nodal marginal zone-B lymphoma non-Hodgkin lymphoma.

The patient was followed up for five years by the urology and oncology departments. In the fourth year of follow-up, the patient had B symptoms (fever, night sweats and weight loss). At the same time, laboratory tests showed haemolytic anaemia; then a new bone marrow biopsy was carried out. The histopathological specimen showed six lymphoid aggregates, constituted by a B-cell population with intra-trabecular predominance and reactivity for CD20 and BCL-2. New thoracic and abdominal computed tomographies were performed without any findings suggestive of extra-prostatic spreading.

Subsequently, a chemotherapy regimen was started on the patient with the following therapeutic scheme: Rituximab 375 mg/m^2^ IV per day, cyclophosphamide 750 mg/m^2^ IV per day, Vincristine 1.4 mg/m^2^ IV dose per day and Prednisone 40 mg/m^2^ on days 1–5 (R-CVP scheme) for 21 days, until he completed six cycles. No signs, symptoms or progression have been recorded.

## Introduction

Lymphoproliferative diseases usually originate in the lymphatic system. However, up to 10% of cases affect the urinary tract, most of which correspond to non-Hodgkin lymphomas (NHL) [1]. Urinary tract involvement occurs in less than 10% of the patients with disseminated disease; however, only 1% of cases present urinary symptoms at the onset of the pathology [1], [2].

The primary involvement of the prostate by a lymphoma is infrequent, accounting for 0.1% overall lymphoproliferative diseases [9]. It usually does not present any symptoms or alter the prostate-specific antigen (PSA) values. The diagnosis is made in an incidental fashion most of the time. Nonetheless, it is an aggressive tumour with high mortality rates. At the moment, there is no single standardized therapeutic guide for its management, requiring several therapeutic strategies.

## Methods

Herein, we present the case of a 68-year-old male patient with a three-year history of severe, progressive, low urinary tract symptoms (LUTS), and 20 points on the International Symptom Scale score. On digital rectal examination, a regular-sized prostate was found, without nodules, with normal consistency, free lateral recesses, no asymmetry and mobile, symmetric seminal vesicles, without masses and a normal sphincter tone. The rest of the physical examination was within normal limits. Additional tests showed a PSA value of 1.4 mg/dL, low and prolonged free uroflowmetry with a post-voidal volume of 64 cc and a prostatic volume of 50 cc measured by transrectal ultrasound. Cystoscopy evaluation revealed a trilobulated prostate with no other significant findings. The patient received alpha-1-blocker therapy without proper cessation of his symptoms.

Because of the clinical presentation, laboratory results, imaging findings and the lack of improvement when pharmaceutical treatment was attempted, a transurethral resection of the prostate was performed. The patient was discharged on the second post-operative day. Removal of the transurethral catheter occurred on the fifth post-operative day, and no complications were reported in the early post-operative period.

## Results

**Figure 1.**
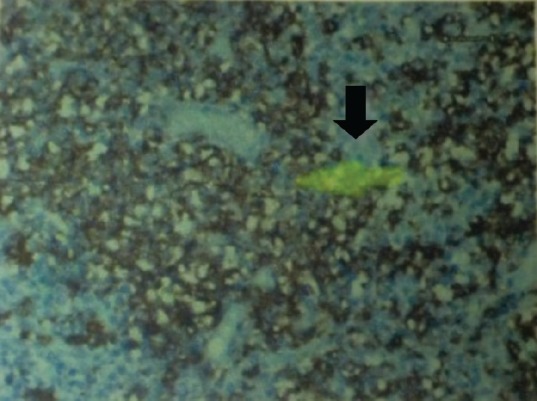
The anatomopathological report showed suspicion of compromise for small-cell neoplasia of possible haematolymphoid strain. Bone marrow cylinders with 60% megakaryocytes cellularity and with presence and evidence of all cell lines. Aggregates of mature lymphocytes of interstitial distribution are indicated by a black arrow.

The anatomopathological report indicates the presence of a haematolymphoid small-cell neoplasia and glandulostromal prostatic hyperplasia (Figure 1). Therefore, it is sent to immunohistochemistry evaluation, reporting an extra-nodal marginal zone-B lymphoma (MALT) NHL, which was supported by a positive CD20 tumoural marker.

**Figure 2.**
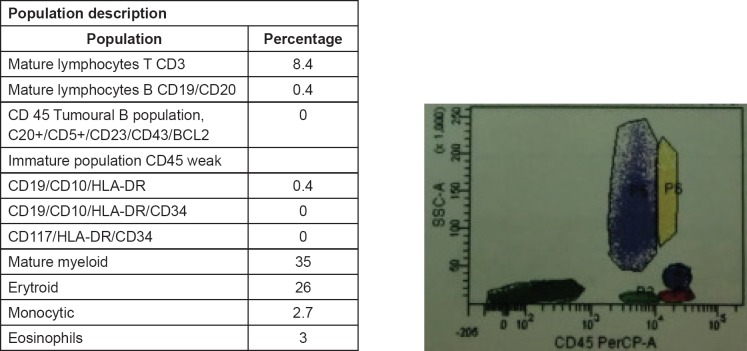
Bone marrow biopsy. Well-differentiated lymphocytic haematolymphoid neoplasm. Flowcytometry does not show infiltration by haematolymphoid neoplasm.

The service of haemato-oncology assessed the patient after two months following the procedure. Cervical, thoracic and abdominal computed tomographies were performed without any findings suggestive of extra-prostatic spreading. Karyotype identified an XY 46 karyotype without pathological alterations. Bone marrow biopsy and flow cytometry were negative to haematolymphoid neoplasia (Figure 2).

The patient was followed up for five years (every six and two months by the urology and the haemato-oncology department, respectively). He did not present LUTS; his I-PSS was five points, with urinary continence and appropriate erectile function without modification in comparison with his functioning before the surgical procedure, and the last control PSA was 0.4 ng/mL. In the fourth year of the follow-up, the patient had B symptoms (fever, night sweats and weight loss). At the same time, laboratory tests showed haemolytic anaemia; then, a new bone marrow biopsy was carried out. The histopathological specimen showed six lymphoid aggregates, constituted by a B-cell population with intra-trabecular predominance and reactivity for CD20 and BCL-2; these findings were the same as those associated with the initial pathology of the prostate. New thoracic and abdominal computed tomographies were performed without any findings suggestive of extra-prostatic spreading.

Subsequently, a chemotherapy regimen was started with the following therapeutic scheme: Rituximab 375 mg/m^2^ IV per day, cyclophosphamide 750 mg/m^2^ IV per day, Vincristine 1.4 mg/m^2^ IV dose per day and Prednisone 40 mg/m^2^ on days 1–5 (R-CVP scheme), for 21 days until he completed six cycles.

The patient finished the sixth chemotherapy cycle with apparent adequate control of the disease and no signs of further progression. The patient showed no B symptoms; complete blood panel showed no anaemia or immature cell lines; the bone marrow biopsy reported no malignant findings; abdominal and thoracic CT evaluation was normal.

## Discussion

A lymphoma is a malignant disease of the reticular system that can affect the adrenal glands and the genitourinary tract in a primary or secondary fashion. Three different types of presentation in the urinary system have been described: primary extra-ganglionic disease, clinical presentation of clinically occult lymph node disease and advanced stage of disseminated lymphoma [3].

Lymphomatous infiltration, both primary and secondary, has been reported on kidneys up to 4.9% [4], ureter [5], bladder up to 0.2% [5] and prostate, penis or testis in less than 0.1% [6–8].

NHL of the prostate represents less than 0.09% of all malignant tumours of the gland [9]. Its diagnosis requires a high degree of clinical suspicion, especially in young males, since there are usually no clinical manifestations; the diagnosis is made incidentally in the setting of other more frequent processes of the prostate gland characterized by obstructive symptoms of the lower urinary tract, such as benign prostatic hyperplasia [10].

MALT-type lymphoma is just 3% of all lymphoma types. It occurs more frequently in women during adult life and represents 5%–10% of all gastric neoplasms. The gastric localization accounts for 70% of the extra-ganglionic involvement. However, MALT-type lymphoma may be seen in lungs, head, neck, thyroid, skin, breast and other sections of the gastrointestinal tract, the prostate being the most remote site with less than 1% of cases [11].

The largest series of cases of lymphomatous infiltration of the prostate reported in the literature (Table 1) show characteristics similar to our patient, regarding age (studied patients were between the fifth and seventh decades of life) and clinical manifestations (they all clinically debuted with LUTS, without urinary retention and a normal PSA in most of them). On digital rectal examination, a varied spectrum of findings has been reported. In most cases, an increase in the consistency of the gland is evidenced, which did not happen in our patient.

Concerning the lymphoma type, MALT-type b-cell NHL is usually managed with surgery and posterior chemotherapy if the bone marrow is involved. In our case, this type of lymphoma was removed surgically. After five years of follow-up, his bone marrow was found to be affected, requiring chemotherapy treatment.

Primary prostatic lymphoma is a rare condition, and it is estimated that there are less than 100 cases described in the world literature to date.

This low incidence has impeded the establishment of a consensus on its management. Notwithstanding, the treatment described includes radiotherapy, chemotherapy and radical prostatectomy (see Table 1). On the other hand, regardless of the stage and histological subtypes, 95% of patients die within 13 months of starting treatment, so it is important to include prostatic lymphoma in the differential diagnoses of a patient with LUTS and no pathological confirmation of its aetiology.

## Conclusions

MALT-type b-cell NHL of the prostate is a rare disease, with a high morbidity and mortality rate, with multiple treatment regimens proposed and without a defined consensus. This type requires chemotherapy once the bone marrow is involved or in the presence of B symptoms.

## Funding

The institution did not fund this study.

## Conflicts of interest

The authors declare no conflicts of interest.

## Ethical approval

All procedures performed in studies involving human participants were in accordance with the ethical standards of the institutional and/or national research committees, and with the 1964 Helsinki declaration and its later amendments or comparable ethical standards. This article does not contain any studies with animals performed by any of the authors.

## Financial disclosure

No financial disclosure.

## Figures and Tables

**Table 1.** Summary of the main series of primary lymphomas in the prostate.

Author	Type of lymphoma	Presentation	Numbers of patients	Management
Bostwick and Mann [12]	Primary	LUTS	7	Mean survival of 14 months
Sarris *et al* [6]	Primary NHL	LUTS	3	CCT
Fukutani *et al* [13]	Primary NHL	LUTS	23	CCT
Wazait *et al* 2003 [14]	Primary B-cell NHL	LUTS	1	TURP, radical RT, complete remission at 3-year follow-up

CCT = combined chemotherapy, traditionally, cyclophosphamide, Adriamycin, vincristine and prednisolone-based regimens were used.

NHL = non-Hodgkin lymphoma.

RT = radiotherapy.

TURP = transurethral resection of the prostate.
